# The Effect of Psychoeducation on Attitudes toward Violence and Risky Behaviors among Refugee Adolescents

**DOI:** 10.3390/bs14070549

**Published:** 2024-06-28

**Authors:** Derya Atik, Ayşe İnel Manav, Edanur Tar Bolacalı

**Affiliations:** 1 Department of Nursing, Faculty of Health Sciences, Osmaniye Korkut Ata University, Osmaniye 80000, Türkiye; ayseinel@gmail.com; 2 First and Emergency Aid Program, Department of Medical Services and Techniques, Vocational School of Health Services, Kırsehir Ahi Evran University, Kırsehir 40100, Türkiye; edanurtar.1107@gmail.com

**Keywords:** adolescent, refugee, psychoeducation, risky behavior, violence

## Abstract

This study was conducted to examine the effect of psychoeducation on attitudes toward violence and risky behaviors among refugee adolescents. This was a randomized controlled experimental study conducted with refugee adolescents (n = 101) studying in a high school in southern Turkey. After psychoeducation, it was determined that there was a significant decrease in the prevalence of antisocial behaviors, alcohol use, suicidal thoughts, unhealthy eating habits, and school dropout thoughts among adolescents according to the subdimensions of the risky behavior scale. Psychoeducation was found to be effective in reducing attitudes toward violence and preventing risky behaviors among refugee adolescents.

## 1. Introduction

Adolescence is defined as a tumultuous process in which hormonal, sexual, social, emotional, and mental changes and developments occur in an individual, starting with rapid physical growth. According to psychosocial development theory, Erikson names this period “Identity-Counter-Identity Confusion” [1]. According to Psychosocial Development Theory, adolescents with “Identity-Counter-Identity Confusion” may be prone to risky behaviors such as violence, substance use, suicide, and nutritional disorders due to inadequately developed coping skills [2,3]. Adolescents take on many roles while questioning their identities in the society in which they live, and as a result of these efforts, they try to find and adopt a suitable identity. In this process, individuals may move away from their families and experience negative emotions such as loneliness, helplessness, insecurity, shame, and guilt [4,5]. Because their coping skills are not sufficiently developed, they may be prone to risky behaviors such as violence, substance abuse, suicide, and nutritional disorders. Adolescents who are still trying to form an identity may be forced to take on an adult role for which they are not ready due to wars and migration. Individuals may also experience feelings such as anxiety about losing their independence, family, and physical integrity; loneliness; and grief [6]. War and migration can make this process even more difficult for adolescents who are in a difficult period of development. Exposure to traumatic events and violence can be associated with various psychological problems in refugee adolescent groups and therefore with a tendency toward violence and risky behavior [7,8,9].

In this dimension, nurses, like other health professionals, have an important role in identifying and preventing the problems and risk factors that threaten the health of refugee adolescents and providing support and consultancy services to adolescents in coping with the long-term effects of the events they experience. Nursing approaches aim to develop individual protective factors among refugee adolescents by conducting psychoeducational programs covering topics such as coping skills, anger management, violence and addiction prevention, risky sexual behaviors in adolescence, assertiveness–timidity, and stress management [10,11]. Psychoeducation programs are based on the “nursing prevention model”, which argues that everyone can be negatively affected by stressful life events, that problems may have many causes, and that any problem or disease may occur as a result; additionally, these programs have positive effects on social functioning and prevention of risky behaviors [12,13,14,15].

Our country is one of the most preferred countries for refugees in its geographical region in terms of both security and political, economic, and religious standards, and refugee adolescents constitute 18% of this group. This rate clearly demonstrates the importance of protecting and improving the health of refugee adolescents in our country [16].

By understanding the impact of psychoeducation on attitudes toward violence and risky behaviors, healthcare professionals and educators can better tailor their support and resources to meet the specific needs of this vulnerable population. Additionally, by shedding light on the potential benefits of psychoeducation, this study may also help to reduce the stigma surrounding mental health issues within refugee communities. It has the potential to empower both adolescents and their families to seek out and engage with mental health resources, ultimately leading to improved overall well-being. For these reasons, we aimed to address this issue and reveal the effect of psychoeducation on attitudes toward violence and risky behaviors among refugee adolescents. This study contributes to the literature, the nursing profession, other healthcare professionals, refugee adolescents and their families, and society.

## 2. Materials and Methods

### 2.1. Study Design

This research was conducted as a randomized controlled, single-blind experimental type to examine the effect of psychoeducation on attitudes toward violence and risky behaviors among refugee adolescents.

### 2.2. Participants

This research was conducted with refugee adolescents studying in a high school in Turkey. The sample of participants was composed of refugee adolescents studying in the specified high school. The participants consisted of individuals who came to Turkey from Syria 2–3 years ago, who did not have a good socioeconomic status, and who largely lived in a camp. Since the high school where this study was conducted is located very close to the camp area, most of the adolescents living in the camp were studying in this high school. The sample of this research consisted of refugee adolescents who studied at this school, met the inclusion criteria for this study (adolescents between the ages of 12 and 18, who are refugees, who can speak and understand Turkish well), and agreed to participate in this research. Power analysis was performed to determine the number of samples, and the power of this study was calculated with the GPower 3.1 program. To determine the number of samples, based on a study similar to our study [17], as a result of the power analysis performed for power = 0.80 and α = 0.05, 90 samples were determined in total, with a minimum of 45 samples in each group.

This study had two study groups: an intervention group and a control group. For the random assignment of the intervention and control groups, the cluster randomization method was applied in the Excel program by a statistician, taking into account the classes of refugee adolescents included in the sample. The sample was selected as follows: “All the classes in which refugee adolescents were studying” were entered into the Excel program, and the classes to be sampled were randomly selected by a statistician [18].

Single blinding was applied by ensuring that the participants were not aware of which group they were in. Considering that there might be losses during the research process, the number of individuals in each group was increased slightly, and this study included 51 adolescents in the intervention group and 50 in the control group.

### 2.3. Interventions

Before this study was conducted, participants were informed about this study, and explanations were given about the operation and duration of the process. The individuals in both groups were evaluated for their attitudes toward violence and risky behavior before and at the end of the interventions. Additionally, at the beginning of this study, a personal information form prepared by the researcher by scanning the literature [19,20] was used to collect information about variables related to individuals. While the psychoeducation intervention prepared in line with the “nursing prevention model” was applied to the adolescents in the intervention group by a specialist psychiatric nurse and a specialist pediatric nurse for 6 months, no application was made to the control group. The training content was created based on the “Coping with Violence and Aggression in Adolescents Psychoeducation Program and Group Psychoeducation” guidelines [21,22]. Psychoeducation modules were created as follows [21,22]:

Module 1: Definition and Characteristics of Adolescence:

- The adolescence period and its characteristics;
- Expected changes during adolescence;
- Common problems during adolescence (physiological, psychosocial, sexual, and reproductive health problems).

Module 2: Risky Behaviors in Adolescence:

- Suicide;
- Violence;
- Risky sexual behavior;
- Accidents;
- Substance abuse.

Module 3: Strengthening Coping Skills:

- Definition and importance of communication;
- Self-awareness;
- I language;
- Assertiveness skill;
- Coping with anger;
- Coping with anxiety;
- Hope–despair;
- Problem-solving skills.

These titles are the titles of each psychoeducation training module. Since this study aimed to determine the effect of psychoeducation on violence and risky behaviors in adolescents, while creating the modules, first, detailed training was given about adolescence (module 1); then, training was given about violence and risky behaviors in adolescence (module 2); and then, training was given about methods of coping with violence and risky behaviors (module 3). Separate presentations were prepared and presented for all topics, and interactive activities were held. The training was conducted in a classroom environment. All modules were composed of training and activities in which adolescents can participate effectively. For the application, the high school where the students were studying was visited once a week. Adolescents were divided into groups of 10–12 people. These groups were administered treatment by specialist pediatric and psychiatric nurses, each lasting approximately 45 min. During this process, no application was made to the control group, and psychoeducation was planned to be applied to the control group after this study. However, due to the earthquake disaster that occurred in our country and because the school where this study was conducted was located in a region affected by the earthquake, psychoeducation could not be applied to the control group after this study.

### 2.4. Instruments

Before the interventions and at the end of the 6th month, the “Adolescents’ Attitudes toward Violence Scale (ATVS)”, “Risky Behaviors Scale (RBS)” and “Personal Information Form” were administered to the participants in both the intervention and control groups.

#### 2.4.1. Personal Information Form

The personal information form was prepared by the researcher by examining the literature on the subject [19,20] and included the personal–sociodemographic characteristics of the adolescents and their families. Considering the personal information that was researched and questioned in previous similar studies, this research aimed to query similar information.

#### 2.4.2. Adolescents’ Attitudes toward Violence Scale

The Adolescents’ Attitudes toward Violence Scale was developed by Çetin (2011). It was developed to measure adolescents’ attitudes toward physical violence. The highest possible score obtained from the scale is 50, and the lowest possible score is 10. A higher score indicates a higher level of violence. The internal consistency reliability coefficient of the scale was found to be 0.85 [23]. The Cronbach’s alpha reliability coefficient of the Adolescents’ Attitudes toward Violence Scale was 0.88 in this study.

#### 2.4.3. Risky Behaviors Scale

The Risky Behaviors Scale was developed by Ergene and Gençtanırım (2014). It is a scale that measures the risky behaviors of secondary school students. The Risky Behaviors Scale is a scale consisting of 36 items and 6 subdimensions. The AB subscale consists of seven items, the AU subscale consists of seven items, the smoking use subscale comprises six items, the suicidal tendency (ST) subscale consists of four items, the eating habit (EH) subscale consists of five items, and the school dropout (SD) subscale consists of seven items. A low score on the scale indicates a low level of risky behavior. The internal consistency coefficient of the scale was found to be 0.91 for the Risky Behavior Scale total score [24]. The Cronbach’s alpha reliability coefficient of the Risky Behaviors Scale was 0.90 in this study.

### 2.5. Statistical Analysis

The data obtained in the present study were analyzed using IBM SPSS Statistics V25.0 (IBM Corp., Armonk, NY, USA), the IBM AMOS statistical package program and R Studio. Descriptive statistics are presented as the number (n), percentage (%), mean ± standard deviation, smallest value (min), largest value (max), and median (median). The chi-square test was used to evaluate categorical data. The normality of the distribution of continuous variables was evaluated with the Kolmogorov–Smirnov/Shapiro–Wilk normality test. When continuous variables met the assumption of normality, an independent sample *t* test was used for two-group comparisons. If the data were not normally distributed, they were evaluated with the Mann-Whitney U test. When the assumption of normality was met, repeated-measures ANOVA was used; when the assumption of normality was not met, the nonparametric F1-LD-F1 test was used for repeated measurements. Bonferroni and Dunn tests were used for post hoc comparisons. In cases where the group*time interaction was significant in repeated measurements, intragroup and intergroup comparisons were evaluated independently. A value of *p* < 0.05 was considered to indicate statistical significance.

### 2.6. Ethical Disclosures

Before this research was started, Ethics Committee approval (decision number 2021\2\23) was obtained from the Osmaniye Korkut University Scientific Research and Publication Ethics Board, and institutional permission was obtained from the Osmaniye Provincial Directorate of National Education. In addition, the purpose of this research was explained to the parents of the individuals participating in this study, and written consent was obtained using the “Informed Volunteer Consent Form”. Verbal consent was obtained from the adolescents.

## 3. Results

The personal characteristics of the individuals included in this research are presented in Table 1.

There was no significant difference in the personal characteristics of the participants between the two groups (*p* ≤ 0.05), and the groups had similar characteristics (Table 1).

The distribution of information about the participants’ living conditions is presented in Table 2.

The median number of years of life in Turkey for individuals in the intervention group was 10.0 (5.0–15.0), and the median for individuals in the control group was 10.0 (7.0–15.0) years (*p* < 0.006) (Table 2). In the present study, 35.3% of the individuals in the intervention group (n = 18) and 34.0% of the individuals in the control group (n = 17) reported that they lost their relatives due to the war (*p* = 0.891). Addictive substance abuse was reported in 9.8% (n = 5) of individuals in the intervention groups and 8.0% (n = 4) of individuals in the control group (*p* = 0.999). Additionally, substance abuse on the part of a relative was reported by 37.3% (19) of the individuals in the intervention groups and 52.0% (n = 26) of the individuals in the control group (*p* = 0.136) (Table 2). Living conditions are important variables as they can have an impact on adolescents’ violent and risky behaviors.

In the evaluation of repeated measurements, repeated-measures ANOVA/nonparametric test F1-LD-F1 design analysis was performed for repeated measures in a factorial design (Table 3). Since group–time interactions were statistically significant for all pre–post-test measurements, the findings are presented not in Table 3 but through independent evaluations within and between groups presented in Table 4.

The scale total score averages/medians are presented in Table 4. Accordingly, between the intervention and control groups, AB was the preliminary total median (*p* = 0.005), ST was the preliminary total median (*p* < 0.001), EH was the final total median (*p* = 0.001), SD was the final total median (*p* = 0.049), and RBS was the preliminary total median (*p* = 0.005). A statistically significant difference was found between the groups in terms of the final total median RBS (*p* = 0.004), the preliminary total median ATVS (*p* = 0.019), and the final total median ATVS (*p* = 0.009) (Table 4).

The median AB pretotal score was 16 (8–26) in the intervention group and 12.5 (7–31) in the control group, and the median AB pretotal score in the intervention group was significantly greater than that in the control group. The median ST pretotal score was 12 (6–18) in the intervention group and 9 (4–20) in the control group, and the median of the intervention group was found to be significantly greater than that of the control group. The mean final total EH score was 10.08 ± 3.52 in the intervention group and 12.82 ± 4.38 in the control group, and the median EH score in the intervention group was significantly lower than that in the control group. The median SD final total score was 7 (7–16) in the intervention group and 9 (7–22) in the control group, and the median of the intervention group was significantly lower than that of the control group. The mean RBS pretotal score was 74.27 ± 15.28 in the intervention group and 64.58 ± 18.65 in the control group, and the median RBS score in the intervention group was significantly greater than that in the control group (*p* < 0.05) (Table 4).

The mean RBS final total score was 57.47 ± 11.8 in the intervention group and 66.86 ± 19.2 in the control group, and the median RBS score in the intervention group was significantly lower than that in the control group. The mean pretotal ATVS score was 28.6 ± 6.9 in the intervention group and 25.2 ± 7.7 in the control group, and the median of the intervention group was found to be significantly greater than that of the control group. The mean ATVS final total score was 22.5 ± 7.6 in the intervention group and 26.7 ± 8.4 in the control group, and the median score of the intervention group was significantly lower than that of the control group (*p* < 0.05) (Table 4).

In the intervention group, the pretotal average AB score (16 [8–26]) was significantly greater than the post-total average AB score (13 [7–19]) (*p* < 0.001). In the intervention group, the median pretotal measurement in the AU subgroup (7.5 [7–22]) was significantly greater than the median post-total measurement (7.0 [7–14]) (*p* = 0.033). In the intervention group, the pretotal measurement median in the ST subgroup (12 [6–18]) was significantly greater than the post-total measurement median (9 [4–17]) (*p* = 0.001). The median pretotal measurement in the EH subgroup (14 [5–22]) was significantly greater than the median post-total measurement (10 [5–19]) in the intervention group (*p* < 0.001). In the intervention group, the median pretotal deviation (12 [7–27]) was significantly greater in the SD subgroup than in the final total interval (7 [7–16]) (*p* < 0.001) (Table 4).

In the intervention group, a significant difference was detected between the pretotal RBS score (74.27 ± 15.28) and the post-total RBS score (57.47 ± 11.8) (*p* < 0.001), and the final average was found to be significantly lower. In the control group, a significant difference was detected between the RBS scale pretotal measurement average (64.58 ± 18.65) and post-total measurement average (66.86 ± 19.2) (*p* = 0.008), and the final measurement average was found to be significantly greater. In the intervention group, a significant difference was detected between the pretotal measurement average (28.6 ± 6.9) and the post-total measurement average (22.5 ± 7.6) on the ATVS (*p* < 0.001), and the final measurement average was found to be significantly lower. In the control group, a significant difference was detected between the pretotal measurement average (25.2 ± 7.7) and post-total measurement average (26.7 ± 8.4) on the ATVS (*p* = 0.026), and the final measurement average was found to be significantly greater (Table 4).

## 4. Discussion

In this study, substance abuse was reported in 9.8% of the individuals in the intervention groups, 8.0% of the individuals in the control group, 37.3% of the relatives of the individuals in the intervention groups, and 52.0% of the relatives of the individuals in the control group. Because these individuals’ coping skills are not sufficiently developed, they may be prone to risky behaviors such as violence, substance abuse, suicide, and nutritional disorders [2,3]. The WHO (2020) reports that violence experienced during adolescence includes a range of actions, from physical fighting and bullying to sexual assault and murder, and that the prevalence of physical fighting and bullying among young people is 40% [25]. Bozzini et al. (2021), as a result of their systematic review of 249 studies related to adolescent risky behaviors, reported that the prevalence of substance addiction, especially alcohol and tobacco use, was 45%, the prevalence of suicide and self-harming behavior was 21.6%, the prevalence of violent or aggressive behavior was 14%, and the prevalence of sexual risky behavior was 11% [26].

In this study, a significant decrease in risky behavior was observed among the adolescents in the intervention group after psychoeducation. In Layne’s (2008) randomized controlled study examining the effectiveness of a school-based group psychotherapy program for adolescents exposed to war, it was concluded that psychoeducation is an effective and efficient method for promoting the recovery of adolescents in postwar environments [12]. Im et al. (2018), in a study of Somali refugee youth living in Kenya, reported that trauma-based psychoeducation had a positive effect on post-traumatic stress disorder symptoms and psychosocial factors [27]. The result of our study’s intervention that created a significant decrease in the level of risky behavior supports the literature on the effect of psychoeducation on risky behavior in adolescents.

In this study, the finding of a significant decrease in attitudes toward violence among the adolescents in the intervention group was similar to the findings of previous studies [5,28]. After the implementation of the Capoeira Angola intervention program developed by Momartin et al. (2019) for refugee adolescents, it was determined that there was a significant decrease in behavioral problems in adolescents in general associated with improvements in interpersonal skills, confidence, respect for oneself and others, self-discipline, and a general sense of responsibility. It was also stated that the initiative developed adolescents in terms of respect, cooperation, and nonviolent communication and interaction [5]. Another study by Baker and Jones (2006) revealed that a curriculum-based program using new music therapy techniques significantly reduced externalizing behaviors at school, such as aggression, overactivity, and hyperactivity, among refugee adolescents [28]. Rasi et al. (2021) examined the effect of an adolescent education program for immigrant Afghan female youth in Iran on their tendency toward high-risk behaviors and determined that there was a significant decrease in the tendency to smoke, substance abuse, alcohol consumption, and unprotected sexual intercourse in the intervention groups. In the same study, they determined that violent tendencies did not significantly differ between the control and intervention groups [29]. Mom et al. (2019) showed that improvements in interpersonal skills, confidence, respect for oneself and others, self-discipline, and a general sense of responsibility were achieved with Capoeira, an Afro-Brazilian martial art applied to young refugees newly settled in Australia. In the same study, a significant decrease in behavioral problems related to these issues was observed in general [4]. In their systematic review, Pottie et al. (2015) found that in many immigrant groups, bullying and peer aggression were significantly greater among first-generation immigrant adolescents who did not speak the official language than among third-generation and native-born adolescents [30].

The sample was drawn from a single high school in southern Turkey, which may limit the generalizability of the findings to other refugee populations. Second, the cultural background and specific experiences of Syrian refugee adolescents may influence the effectiveness of the psychoeducation program, and results may differ in other cultural contexts. Additionally, this study did not assess the long-term impact of the intervention, and further research is needed to determine the sustainability of the observed behavioral changes.

To address these limitations, future research should consider conducting multi-center studies involving refugee adolescents from diverse geographical and cultural backgrounds to enhance the generalizability of the findings. Longitudinal studies are also recommended to assess the long-term effects of psychoeducation on violent and risky behaviors. Furthermore, qualitative research could provide deeper insights into the personal experiences of refugee adolescents and how they perceive and engage with psychoeducation programs.

## 5. Conclusions

The final total score on the risky behavior scale and the final total score on the attitude toward violence scale were significantly lower for the adolescents in the intervention group than for those in the control group. Among the adolescents’ risky behavior scale subdimensions, there was a significant decrease in antisocial behavior, alcohol use, suicidal ideation, unhealthy eating habits, and school dropout thoughts. It was determined that there was a significant decrease in the attitudes of the adolescents in the intervention group toward risky behavior and violence. It has been determined that psychoeducation is an effective intervention for reducing risky behaviors and improving attitudes toward violence among refugee adolescents.

## Figures and Tables

**Table 1:** Distribution of individuals’ personal characteristics in groups.

		Group		Total	Test Statistics *p* Value
		Control	Intervention		
Age	Average Median	14.9 ± 0.8 15.0 [13.0–17.0]	14.9 ± 0.7 15.0 [14.0–16.0]	14.9 ± 0.8 15.0 [13.0–17.0]	U = 1193.0 *p* = 0.540
Gender	Girl	19 (38.0)	20 (39.2)	39 (38.6)	X^2^ = 0.016 *p* = 0.900
	Male	31 (62.0)	31 (60.8)	62 (61.4)	
	Total	50 (100)	51 (100)	101 (100)	
Number of siblings	One	1 (2.0)	0 (0.0)	1 (1.0)	X^2^ = 2.053 *p* = 0.562
	Two	7 (14.0)	5 (9.8)	12 (11.9)	
	Three	13 (26.0)	11 (21.6)	24 (23.8)	
	Four and above	29 (58.0)	35 (68.6)	64 (63.4)	
	Total	50 (100)	51 (100)	101 (100)	
Order of birth	One	15 (30.0)	16 (31.4)	31 (30.4)	X^2^ = 1.639 *p* = 0.651
	Two	15 (30.0)	15 (29.4)	30 (29.7)	
	Three	13 (26.0)	9 (17.6)	22 (21.8)	
	Four and above	7 (14.0)	11 (21.6)	18 (17.8)	
	Total	50 (100)	51 (100)	101 (100)	
Mother’s education level	Not literate	1 (2.0)	7 (13.7)	8 (7.9)	X^2^ = 7.285 *p* = 0.122
	Primary school graduate	7 (14.0)	10 (19.6)	17 (16.8)	
	Secondary school graduate	28 (56.0)	26 (51.0)	54 (53.5)	
	High school graduate	13 (26.0)	8 (15.7)	21 (20.8)	
	Graduated from a university	1 (2.0)	0 (0.0)	1 (1.0)	
	Total	50 (100)	51 (100)	101 (100)	
Father’s education level	Not literate	4 (8.0)	7 (13.7)	11 (10.9)	X^2^ = 3.903 *p* = 0.419
	Primary school graduate	11 (22.0)	7 (13.7)	18 (17.8)	
	Secondary school graduate	23 (46.0)	26 (51.0)	49 (48.5)	
	High school graduate	8 (16.0)	10 (19.6)	18 (17.8)	
	Graduated from a university	4 (8.0)	1 (2.0)	5 (5.0)	
	Total	50 (100)	51 (100)	101 (100)	
Mother’s working status	Worker	14 (28.0)	11 (21.6)	25 (24.8)	X^2^ = 1.684 *p* = 0.431
	Not working	35 (70.0)	40 (78.4)	75 (74.3)	
	Not alive	1 (2.0)	0 (0.0)	1 (1.0)	
	Total	50 (100)	51 (100)	101 (100)	
Father’s working status	Worker	44 (88.0)	42 (82.4)	86 (85.1)	X^2^ = 2.237 *p* = 0.525
	Not working	3 (6.0)	6 (11.8)	9 (8.9)	
	Retired	0 (0.0)	1 (2.0)	1 (1.0)	
	Not alive	3 (6.0)	2 (3.9)	5 (5.0)	
	Total	50 (100)	51 (100)	101 (100)	
Living situation of parents	Yes	44 (88.0)	49 (96.1)	93 (92.1)	X^2^ = 2.259 *p* = 0.160
	No	6 (12.0)	2 (3.9)	8 (7.9)	
	Total	50 (100)	51 (100)	101 (100)	
Economic income perception status	Good	3 (6.0)	6 (11.8)	9 (8.9)	X^2^ = 3.899 *p* = 0.144
	Middle	35 (70.0)	26 (51.0)	61 (60.4)	
	Bad	12 (24.0)	19 (37.3)	31 (30.7)	
	Total	50 (100)	51 (100)	101 (100)	

X^2^: chi-square test statistic, U: Mann-Whitney U test statistic, *p* < 0.05 significance level.

**Table 2:** Distribution of participants’ information about living conditions in groups.

		Group		Total	Test Statistics *p* Value
		Control	Intervention		
Duration lived in Turkey (years)	Average Median	10.5 ± 1.2 10.0 [7.0–15.0]	9.5 ± 2.3 10.0 [5.0–15.0]	10.0 ± 1.9 10.0 [5.0–15.0]	U = 881.0 *p* = 0.006
Living place	House	10 (20.0)	11 (21.6)	21 (20.8)	X^2^ = 0.038 *p* = 0.846
	Camp	40 (80.0)	40 (78.4)	80 (79.2)	
	Total	50 (100)	51 (100)	101 (100)	
The person you live with	Mother	4 (8.0)	2 (3.9)	6 (5.9)	X^2^ = 2.857 *p* = 0.281
	Father	4 (8.0)	1 (2.0)	5 (5.0)	
	Mother, father, siblings	42 (84.0)	48 (94.1)	90 (89.1)	
	Total	50 (100)	51 (100)	101 (100)	
Changes in social relations after coming to Turkey	Changed Positive	2 (4.0)	3 (5.9)	5 (5.0)	X^2^ = 0.313 *p* = 0.999
	Changed Negatively	44 (88.0)	43 (84.3)	87 (86.1)	
	Unchanged	4 (8.0)	5 (9.8)	9 (8.9)	
	Total	50 (100)	51 (100)	101 (100)	
Do you have a relative who died or disappeared due to war?	Yes	17 (34.0)	18 (35.3)	35 (34.7)	X^2^ = 0.019 *p* = 0.891
	No	33 (66.0)	33 (64.7)	66 (65.3)	
	Total	50 (100)	51 (100)	101 (100)	
Is there anyone whose safety you are worried about where you come from?	Yes	23 (46.0)	27 (52.9)	50 (49.5)	X^2^ = 0.487 *p* = 0.485
	No	27 (54.0)	24 (47.1)	51 (50.5)	
	Total	50 (100)	51 (100)	101 (100)	
Do you use cigarettes, alcohol, or addictive substances?	Yes	4 (8.0)	5 (9.8)	9 (8.9)	X^2^ = 0.101 *p* = 0.999
	No	46 (92.0)	46 (90.2)	92 (91.1)	
	Total	50 (100)	51 (100)	101 (100)	
Do your relatives use cigarettes, alcohol or addictive substances?	Yes	26 (52.0)	19 (37.3)	45 (44.6)	X^2^ = 2.222 *p* = 0.136
	No	24 (48.0)	32 (62.7)	56 (55.4)	
	Total	50 (100)	51 (100)	101 (100)	

X^2^: chi-square test statistic, U: Mann-Whitney U test statistic, *p* < 0.05 significance level.

**Table 3:** Evaluation of repeated measurements.

	Intervention Group		Control Group		Test Statistics *p*-Value	Partial eta Square
	$\bar{X}\pm SS$	$\tilde{X}$ [min–max]	$\bar{X}\pm SS$	$\tilde{X}$ [min–max]		
**AB pretotal**	16.33 ± 4.72	16 [8–26]	14.1 ± 6.05	12.5 [7–31]	Group: WTS = 0.597 *p* = 0.439	0.001
**AB final total**	12.43 ± 3.44	13 [7–19]	14.6 ± 5.5	14 [7–30]	Time: WTS = 10.716 *p* = 0.001	0.146
					Group × Time: WTS = 26.28 *p* < 0.001	0.223
**AU pretotal**	9.37 ± 3.93	7.5 [7–22]	8.54 ± 3.67	7 [7–25]	Group: WTS = 0.261 *p* = 0.609	0.001
**AU final total**	8.08 ± 2.29	7.0 [7–14]	9.12 ± 4.16	7 [7–25]	Time: WTS = 0.488 *p* = 0.484	0.011
					Group × Time: WTS = 6.702 *p* = 0.009	0.071
**Smoking pretotal**	9.37 ± 4.31	8 [6–23]	9.08 ± 5.05	6 [6–26]	Group: WTS = 0.010 *p* = 0.917	0.005
**Smoking final total**	8.24 ± 4	6 [6–26]	9.7 ± 5.29	6 [6–26]	Time: WTS = 0.567 *p* = 0.451	0.004
					Group × Time: WTS = 6.534 *p* = 0.010	0.045
**ST pretotal**	12.25 ± 3.57	12 [6–18]	9.16 ± 4.31	9 [4–20]	Group: WTS = 4.663 *p* = 0.030	0.039
**ST final total**	9.57 ± 3.85	9 [4–17]	9.8 ± 4.54	10.5 [4–20]	Time: WTS = 7.026 *p* = 0.008	0.065
					Group × Time: WTS = 19.73 *p* < 0.001	0.155
**EH pretotal**	14.37 ± 3.84	14 [5–22]	12.94 ± 4.81	13 [5–21]	Group: WTS = 0.768 *p* = 0.380	0.008
**EH final total**	10.08 ± 3.52	10 [5–19]	12.82 ± 4.38	13 [5–21]	Time: WTS = 30.90 *p* < 0.001	0.243
					Group × Time: WTS = 29.71 *p* < 0.001	0.223
**SD pretotal**	12.57 ± 5.33	12 [7–27]	10.76 ± 4.3	9 [7–22]	Group: WTS = 0.016 *p* = 0.898	0.001
**SD final total**	9.08 ± 2.77	7 [7–16]	10.82 ± 4.48	9 [7–22]	Time: WTS = 20.48 *p* < 0.001	0.144
					Group × Time: WTS = 18.02 *p* < 0.001	0.152
**RBS pretotal**	74.27 ± 15.28	71 [44–115]	64.58 ± 18.65	60.5 [37–123]	Group: F = 0.003 *p* = 0.959	0.001
**RBS final total**	57.47 ± 11.8	56 [38–84]	66.86 ± 19.2	63 [40–123]	Time: F = 28.831 *p* < 0.001	0.226
					Group × Time: F = 49.776 *p* < 0.001	0.335
**ATVS pretotal**	28.6 ± 6.9	29 [14–45]	25.2 ± 7.7	27 [10–41]	Group: F = 0.081 *p* = 0.777	0.001
**ATVS final total**	22.5 ± 7.6	21 [10–39]	26.7 ± 8.4	27 [10–49]	Time: F = 9.033 *p* = 0.003	0.084
					Group × Time: F = 23.930 *p* < 0.001	0.195

WTS: Wald-type statistic. F: repeated-measures ANOVA test statistic. *p* < 0.05 significance level. In the evaluation of repeated measurements, repeated-measures ANOVA or nonparametric tests for repeated measurements in the factorial design F1-LD-F1 design analysis were performed. AB: Antisocial Behavior. AU: Alcohol Use. ST: Suicidal Tendencies. EH: Eating Habits. SD: School Dropout. ATVS: Adolescents’ Attitudes toward Violence Scale. RBS: Risky Behaviors Scale.

**Table 4:** Comparisons of scale score averages/medians within and between groups.

	Intervention Group			Control Group			Test Statistics *p* Value
	$\bar{X}\pm SS$	$\tilde{X}[min–max]$	Test Statistics *p* Value	$\bar{X}\pm SS$	$\tilde{X}[min–max]$	Test Statistics *p* Value	
**AB pretotal**	16.33 ± 4.72	16 [8–26]	W = −4.195 *p* < 0.001	14.1 ± 6.05	12.5 [7–31]	W = −1.879 *p* = 0.060	U = 861.5 *p* = 0.005
**AB final total**	12.43 ± 3.44	13 [7–19]		14.6 ± 5.5	14 [7–30]		U = 1039.5 *p* = 0.108
**AU pretotal**	9.37 ± 3.93	7.5 [7–22]	W = −2.136 *p* = 0.033	8.54 ± 3.67	7 [7–25]	W = −1.841 *p* = 0.066	U = 1085 *p* = 0.101
**AU final total**	8.08 ± 2.29	7.0 [7–14]		9.12 ± 4.16	7 [7–25]		U = 1183.5 *p* = 0.418
**Smoking pretotal**	9.37 ± 4.31	8 [6–23]	W = −1.547 *p* = 0.122	9.08 ± 5.05	6 [6–26]	W = −1.663 *p* = 0.096	U = 1124 *p* = 0.264
**Smoking final total**	8.24 ± 4	6 [6–26]		9.7 ± 5.29	6 [6–26]		U = 1095.5 *p* = 0.180
**ST pretotal**	12.25 ± 3.57	12 [6–18]	W = −3.453 *p* = 0.001	9.16 ± 4.31	9 [4–20]	W = −1.787 *p* = 0.074	U = 721.5 *p* < 0.001
**ST final total**	9.57 ± 3.85	9 [4–17]		9.8 ± 4.54	10.5 [4–20]		U = 1268.5 *p* = 0.965
**EH pretotal**	14.37 ± 3.84	14 [5–22]	W = −4.793 *p* < 0.001	12.94 ± 4.81	13 [5–21]	W = −0.286 *p* = 0.775	U = 1046.5 *p* = 0.120
**EH final total**	10.08 ± 3.52	10 [5–19]		12.82 ± 4.38	13 [5–21]		t = 3.471 *p* = 0.001
**SD pretotal**	12.57 ± 5.33	12 [7–27]	W = −3.847 *p* < 0.001	10.76 ± 4.3	9 [7–22]	W = −0.387 *p* = 0.699	U = 1026 *p* = 0.088
**SD final total**	9.08 ± 2.77	7 [7–16]		10.82 ± 4.48	9 [7–22]		U = 999.5 *p* = 0.049
**RBS pretotal**	74.27 ± 15.28	71 [44–115]	t = 6.583 *p* < 0.001	64.58 ± 18.65	60.5 [37–123]	t = −2.760 *p* = 0.008	t = −2.860 *p* = 0.005
**RBS final total**	57.47 ± 11.8	56 [38–84]		66.86 ± 19.2	63 [40–123]		t = 2.968 *p* = 0.004
**ATVS pretotal**	28.6 ± 6.9	29 [14–45]	t = 4.368 *p* < 0.001	25.2 ± 7.7	27 [10–41]	t = −2.289 *p* = 0.026	t = −2.387 *p* = 0.019
**ATVS final total**	22.5 ± 7.6	21 [10–39]		26.7 ± 8.4	27 [10–49]		t = 2.648 *p* = 0.009

t: Student’s *t* test statistic. U: Mann-Whitney U test statistic. W: Wilcoxon test statistic. *p* < 0.05 significance level. AB: Antisocial Behavior. AU: Alcohol Use. ST: Suicidal Tendencies. EH: Eating Habits. SD: School Dropout. ATVS: Adolescents’ Attitudes toward Violence Scale. RBS: Risky Behaviors Scale.

## Data Availability

The data can be obtained from the authors.
